# Gut microbial signatures of advanced hepatocellular carcinoma and their potential diagnostic value

**DOI:** 10.3389/fmicb.2026.1760859

**Published:** 2026-02-02

**Authors:** Yan Wang, Zhen Yang, Chuang Liu, Yufeng Liu, Zhongyuan Bai, Wentao Miao, Tiantian Zhang, Yan Wang, Xiang Li, Zhiyong Lai, Jun Xu

**Affiliations:** 1 First Clinical Medical College, Shanxi Medical University, Taiyuan, China; 2 Microbiological Laboratory of Ophthalmology, Shanxi Eye Hospital, Taiyuan, China; 3 Fifth Clinical Medical College, Shanxi Medical University, Taiyuan, China; 4 Assisted Reproduction Center, First Hospital of Shanxi Medical University, Taiyuan, China; 5 Department of Hepatobiliary Surgery and Liver Transplantation Center, First Hospital of Shanxi Medical University, Taiyuan, China; 6 Shanxi Provincial Key Laboratory for Digestive Diseases and Organ Transplantation, First Hospital of Shanxi Medical University, Taiyuan, China; 7 Department of Biliopancreatic Surgery, First Hospital of Shanxi Medical University, Taiyuan, China

**Keywords:** advanced hepatocellular carcinoma, biomarkers, Enterococcus, gut microbiome, liver cirrhosis, machine learning, noninvasive diagnosis

## Abstract

**Background:**

Hepatocellular carcinoma (HCC) is a prevalent and lethal malignancy worldwide. Gut microbiota play crucial roles in liver disease progression and may offer noninvasive diagnostic value, yet microbial signatures specific to advanced HCC remain unclear.

**Methods:**

Seventy-six participants, including early-stage HCC (HCC12), advanced HCC (HCC34), liver cirrhosis (LC), and healthy controls (CG), were prospectively enrolled. Fecal samples underwent 16S rRNA sequencing to characterize microbial diversity and community composition. Differential taxa were identified using Kruskal–Wallis tests, linear discriminant analysis effect size (LEfSe), and zero-inflated negative binomial regression (ZINB). Machine learning models were constructed using clinical features, representative microbiota, and their combination. External validation was performed using 74 published HCC cases.

**Results:**

Advanced HCC exhibited reduced microbial richness and diversity, accompanied by substantial community structure alterations. *Enterococcus*, *Enterococcaceae*, *Enterobacteriaceae*, and *Escherichia–Shigella* were enriched in HCC34, whereas *Ruminococcus* and *Blautia* were depleted. These taxa correlated strongly with liver injury markers and HCC-specific biomarkers. The extreme gradient boosting model showed high diagnostic potential when using either clinical or microbial features alone, while the combined model achieved improved accuracy (AUC = 1.0 in the primary test set). External validation supported the good generalizability of the model (AUC = 1.0 in the external cohort). Feature importance analysis identified *Enterococcus* as the most influential discriminator of advanced HCC.

**Conclusion:**

This study reveals distinct gut microbial signatures associated with advanced HCC and suggests that *Enterococcus* may serve as a potentially important microbial marker linked to disease severity. Integrating gut microbiota profiling with clinical features may offer a promising noninvasive strategy for the accurate identification of advanced HCC and provides hypothesis-generating insights for microbiome-based therapeutic interventions.

## 1 Introduction

According to the latest data from the International Agency for Research on Cancer (GLOBOCAN 2022), liver and intrahepatic bile duct malignancies rank sixth in global cancer incidence, with approximately 866,000 new cases each year and an age-standardized incidence rate of 8.6 per 100,000 individuals. Liver cancer accounts for an estimated 759,000 deaths annually, making it the third leading cause of cancer-related mortality worldwide (Bray et al., 2024). Hepatocellular carcinoma (HCC) is the most common type of liver cancer, representing nearly 80% of all cases (Rumgay et al., 2022). China has the highest global burden of HCC, with both incidence and mortality accounting for nearly half of the worldwide total. Due to its insidious clinical presentation and the lack of precise biomarkers, many patients are diagnosed at an advanced stage. Currently, various therapeutic options are available for HCC at different stages, including surgical resection, local ablation, locoregional interventions, and systemic therapy. Selecting an individualized treatment strategy depends critically on the accurate staging of HCC. Combined diagnostic approaches integrating alpha-fetoprotein (AFP), alpha-fetoprotein *Lens culinaris* agglutinin 3 (AFP-L3), and prothrombin induced by the absence of vitamin K or antagonist-II (Pivka II) with imaging modalities are now widely used and have significantly improved diagnostic sensitivity and specificity (Johnson et al., 2014; Berhane et al., 2016; Best et al., 2016; Tayob et al., 2023). At the molecular level, circulating tumor cells (CTCs), cell-free DNA (cfDNA), and circulating tumor DNA (ctDNA) have shown substantial promise in early detection, diagnosis, prognosis prediction, disease monitoring, and therapeutic response assessment in HCC (Chan et al., 2024). However, current diagnostic methods still face limitations, including suboptimal specificity, missed detection of small lesions, high cost, low detection rates, and limited sensitivity. Therefore, continued exploration of diverse biological markers for the precise diagnosis of HCC remains critically important.

With the expanding application of microbiome research, the relationship between gut microbiota and malignant tumors has gained increasing attention. Gut microbial dysbiosis can promote tumor initiation and progression through multiple mechanisms (Schwabe and Greten, 2020; El Tekle and Garrett, 2023). Overgrowth of pathogenic bacteria disrupts the intestinal mucosal barrier and triggers sustained inflammatory responses, which, in turn, drive aberrant cell proliferation and elevate cancer risk (Kim and Lee, 2021). Certain pathogenic taxa, such as *Clostridium* and *Escherichia*, produce carcinogenic metabolites, including nitrosamines and secondary bile acids, that directly damage epithelial DNA and induce gene mutations. The gut microbiota also play a crucial role in hepatocarcinogenesis through lipopolysaccharide (LPS) and its receptor toll-like receptor 4 (TLR4) signaling pathways (Dapito et al., 2012; Roje et al., 2024). In contrast, specific beneficial microbes and their metabolites exert antitumor effects (Redman et al., 2014). For instance, *Bifidobacteria* and *Lactobacillus* secrete short-chain fatty acids (SCFA) (e.g., acetate and propionate) that regulate intestinal pH, inhibit pathogen overgrowth, protect the mucosal barrier, and directly suppress tumor cell proliferation while inducing apoptosis (Lee and Hase, 2014). Additionally, gut microbes shape both innate and adaptive immunity by enhancing immune cell activation and improving antitumor responses (Zhou et al., 2021). As key modulators of cancer immunity, gut microbiota dynamically influence therapeutic responsiveness through bidirectional interactions with the host immune system (Yu and Schwabe, 2017).

Given the pivotal role of gut microbiota in tumor biology, increasing efforts have focused on leveraging microbial signatures as biomarkers for cancer diagnosis, disease progression, and prognosis prediction (Gok Yavuz et al., 2023). In HCC, the gradual transition from chronic hepatitis to cirrhosis and eventually to hepatocellular carcinoma is accompanied by progressive alterations in the gut microbiome, making microbial profiling clinically informative for distinguishing stages of liver disease (Liu and Yang, 2023). In this study, we characterized gut microbial features across early-stage HCC, advanced HCC, liver cirrhosis, and healthy individuals using 16S rDNA sequencing. By integrating microbial signatures with commonly used clinical serological markers, we developed machine learning models to identify advanced HCC and validated their performance using an external dataset. This work provides preliminary insights into the gut microbial characteristics of advanced HCC and highlights their potential value in clinical diagnosis.

## 2 Materials and methods

### 2.1 Study population and design

We prospectively and randomly enrolled 38 patients with HCC, including 18 with stage I–II (HCC12) and 20 with stage III–IV disease (HCC34), as well as 19 patients with liver cirrhosis (LC) and 19 healthy adults (CG), who presented to the First Hospital of Shanxi Medical University between September 2023 and July 2024 for diagnosis and/or clinical management. Fresh fecal samples from patients with liver cirrhosis and HCC were collected on the first day of admission, prior to any therapeutic intervention. Samples were rapidly frozen in liquid nitrogen for 15 min, and subsequently stored at −80 °C. Clinical features, including medical history, laboratory findings, clinical presentation, and disease classification, were retrieved from electronic medical records and the laboratory information system. The diagnosis of liver cirrhosis was established based on hematological tests combined with imaging or histopathology. HCC diagnosis was confirmed using AFP, AFP-L3, and Pivka-II in combination with at least two imaging modalities or histopathological examination. HCC staging followed the latest China liver cancer staging (CNLC) system (Zhou et al., 2025), which incorporates performance status scoring to comprehensively assess treatment tolerance. Staging information for enrolled HCC patients is summarized in Table 1.

**Table 1:** Staging criteria according to the China liver cancer staging (CNLC).

Basis for liver cancer staging	HCC12 (*n* = 18)	HCC34 (*n* = 20)
Performance status	1.667 (0.4851)	2.3 (0.5712)
Extrahepatic metastas	0 (0%)	5 (25%)
Imaging-detected vascular tumor thrombus	0 (0%)	11 (55%)
Tumor number	1.444 (0.7048)	1.7 (1.174)
Maximum tumor diameter, cm	4.644 (3.479)	6.79 (5.525)

CNLC	1a	1b	2a	2b	3a	3b	4
Num	11	1	6	0	5	2	13

Data are *n* (%) or mean (SD).

All participants had no constipation, hematochezia, diarrhea, dyspepsia, or other gastrointestinal symptoms. None had taken antibiotics or acid suppressants within the preceding month. None of the patients with HCC had undergone surgical resection, local ablation, transarterial therapies, systemic therapy, or immunotherapy at the time of sample collection. No patient had a history of systemic malignancies other than HCC.

### 2.2 16S rRNA amplicon sequencing

Genomic DNA was extracted from fecal samples via the Magnetic Soil and Stool DNA Kit (TianGen, China; Catalog No. DP712) following the manufacturer’s instructions. DNA quality was assessed using 1% agarose gel electrophoresis, and qualified samples were diluted with nuclease-free sterile water to a final concentration of 1 ng/μL. The V3–V4 hypervariable regions of the 16S rRNA gene were amplified using primers 341F (CCTAYGGGRBGCASCAG) and 806R (GGACTACNNGGGTATCTAAT). Each PCR reaction contained 15 μL of Phusion^®^ High-Fidelity PCR Master Mix (New England Biolabs), 0.2 μM of each primer, and 10 ng of genomic DNA. The thermal cycling protocol consisted of an initial denaturation at 98 °C for 1 min; 30 cycles of 98 °C for 10 s, 50 °C for 30 s, and 72 °C for 30 s; followed by a final extension at 72 °C for 5 min. PCR amplicons were purified using magnetic beads, and target fragments were recovered via the Universal DNA Purification Kit (TianGen, China; Catalog No. DP214). Library preparation was performed via the NEBNext^®^ Ultra^™^ II FS DNA PCR-Free Library Prep Kit (New England Biolabs, United States; Catalog No. E7430L). Libraries were quantified via Qubit 2.0 fluorometry and qPCR prior to sequencing on the NovaSeq 6000 platform with a paired-end 250 bp strategy.

Raw sequencing data were processed by merging paired-end reads, performing stringent quality filtering, and removing chimeric sequences to obtain high-quality effective tags. The DADA2 module in quantitative insights into microbial ecology 2 (QIIME2, v2022.02) was used for denoising to generate amplicon sequence variants (ASVs) and a feature table. Taxonomic annotation was conducted via the classify-sklearn algorithm in QIIME2 against the SILVA 138.1 reference database.

### 2.3 Bioinformatic analysis

Multiple sequence alignment of all ASV representative sequences was conducted in QIIME2 to infer phylogenetic relationships. To minimize sequencing depth bias, all samples were rarefied to the minimum sequencing depth observed across the dataset. Alpha diversity indices were calculated via QIIME2 and visualized with R (v4.0.3). Beta diversity was assessed based on weighted and unweighted UniFrac distances. Beta diversity heatmaps, and non-metric multi-dimensional scaling (NMDS) analyses were performed via QIIME2, R (v4.0.3), and Perl (v5.26.2). Microbial functional prediction was performed via Tax4Fun (v0.3.1).

### 2.4 Machine learning model construction

Based on the top 15 microbial families and genera identified by the Kruskal–Wallis test in the microbiome analysis, together with clinical features from 38 HCC patients collected at our center, we randomly divided the dataset into a training set and a test set at a 7:3 ratio. Using the scikit-learn package in Python (v3.5.0), we constructed three ensemble machine learning models: random forest (RF), gradient boosting decision tree (GBDT), and extreme gradient boosting (XGB). Model performance was evaluated across multiple metrics, including accuracy, recall, *F*_1_-score, Matthews correlation coefficient (MCC), and area under the ROC curve (AUC), to identify the optimal classifier. For the final selected model, we further assessed stability and generalizability using 5-fold cross-validation (CV) and 200 bootstrap resampling iterations. SHAP analysis was then applied to quantify the contribution of each feature to the model’s classification output and to identify key predictive features based on importance ranking. Finally, external validation was performed using 74 published HCC cases (Zhang et al., 2021), with additional evaluation using 10-fold CV and 200 bootstrap iterations. Because *Enterococcus* and *Bacilli* were not directly available in the external dataset, they were substituted with *Enterococcaceae* and *Lachnospiraceae*, respectively. This substitution does not imply strict taxonomic equivalence and was applied to enable approximate feature alignment across datasets. In addition, and missing values for three features (Pivka II, *Pseudomonas*, and *Moraxellaceae*) were imputed using the mean values from the training set.

### 2.5 Statistical analysis

Clinical data were analyzed via the *t*-test, Kolmogorov–Smirnov test, analysis of variance (ANOVA), Welch’s ANOVA, or the Kruskal–Wallis test, as appropriate. No clinical data were missing. Differences in gut microbial communities were evaluated via zero-inflated negative binomial regression (ZINB), Kruskal–Wallis rank-sum tests, and linear discriminant analysis effect size (LEfSe, v1.1.01). All analyses and visualizations were performed via QIIME2 (v2022.02), Perl (v5.26.2), Python, and R (v3.4.3). A *p*-value <0.05 was considered statistically significant, and multiple comparisons were adjusted via the false discovery rate (FDR) according to the Benjamini–Hochberg procedure.

## 3 Result

### 3.1 Characteristics of the study population

A total of 76 participants were ultimately recruited for this study. The clinical features of all participants are summarized in Table 2. Males predominated in all groups, consistent with the known epidemiological features of cirrhosis and HCC. To ensure that the healthy participants were free of any disease, their mean age was significantly lower than that of the other groups, averaging 41.37 years. Alanine aminotransferase (ALT) levels in the HCC12 group were higher than those in the LC group, whereas aspartate aminotransferase (AST) levels did not differ among the HCC12, HCC34, and LC groups. Compared with the HCC12 group, the HCC34 group exhibited significantly lower albumin (ALB) and cholinesterase (ChE) levels, along with significantly higher total bilirubin (TBil) levels and Child–Pugh scores. Furthermore, the lg(Pivka II) values were significantly higher in the HCC34 group than in the HCC12 group.

**Table 2:** Clinical features of the study participants.

Clinical features	HCC12 (*n* = 18)	HCC34 (*n* = 20)	LC (*n* = 19)	CG (*n* = 19)	*p*-value
Primary disease					
Hepatitis B	14 (77.78%)	13 (65%)	15 (78.95%)	/	/
Alcohol	3 (16.67%)	4 (20%)	3 (15.79%)	/	/
Hepatitis C	1 (5.55%)	2 (10%)	1 (5.26%)	/	/
Else	0 (0%)	1 (5%)	0 (0%)	/	/
Features					
Gender (male)	15 (83.33%)	16 (80%)	15 (78.95%)	12 (63.16%)	0.5395
Age, years	52.22 (11.34)^a^	53.4 (10.15)^a^	52.37 (9.511)^a^	41.37 (9.459)	0.0011
BMI, kg/m^2^	23.16 (4.328)	23.43 (3.607)	24.34 (3.43)	/	0.6144
ALT, U/L	54.39 (54.59)^b^	40.2 (33.07)	33.53 (34.59)	/	0.0356
AST, U/L	59.33 (61.15)	77.4 (70.97)	66.63 (111.8)	/	0.1148
ALB, g/L	36.28 (4.627)^c^	31.99 (5.717)	33.28 (5.541)	/	0.0405
TBil, μmol/L	26.46 (16.07)^c^	91.39 (91.18)	46.01 (33.7)	/	0.0065
ChE, U/L	5,066 (2061)^c^	3,370 (1500)	3,839 (2023)	/	0.0262
ALP, U/L	99.61 (24)	136.7 (63)	96.79 (42.6)	/	0.0549
Child–Pugh score	7.278 (1.406)^c^	9.45 (2.762)	8.526 (2.27)	/	0.0084
PT-S, s	16.34 (2.194)	19.44 (6.261)	17.75 (4.129)	/	0.1232
lg(AFP, ng/mL)	1.121 (0.8789)	1.543 (0.9836)	/	/	0.1731
lg(AFP-L3, ng/mL)	0.03979 (0.6125)	0.8047 (1.307)	/	/	0.3841
lg(Pivka II, ng/mL)	1.589 (0.8428)^c^	2.829 (1.157)	/	/	0.0006

Data are *n* (%) or mean (SD). BMI, body mass index; ALT, alanine aminotransferase; AST, aspartate aminotransferase; ALB, albumin; TBil, total bilirubin; ChE, cholinesterase; ALP, alkaline phosphatase; PT-S, prothrombin time-seconds; AFP, alpha-fetoprotein; AFP-L3, alpha-fetoprotein *Lens culinaris* agglutinin 3; Pivka II, prothrombin induced by the absence of vitamin K or antagonist-II. For normally distributed variables with homogeneity of variance, one-way ANOVA or *t*-test was used; when variance was unequal, the Brown–Forsythe ANOVA or Kolmogorov–Smirnov was applied. For non-normally distributed data, the Kruskal–Wallis test was performed. ^a^*p* < 0.05 vs. CG; ^b^*p* < 0.05 vs. LC; ^c^*p* < 0.05 vs. HCC34.

### 3.2 Gut microbial diversity in HCC

**Figure 1:**
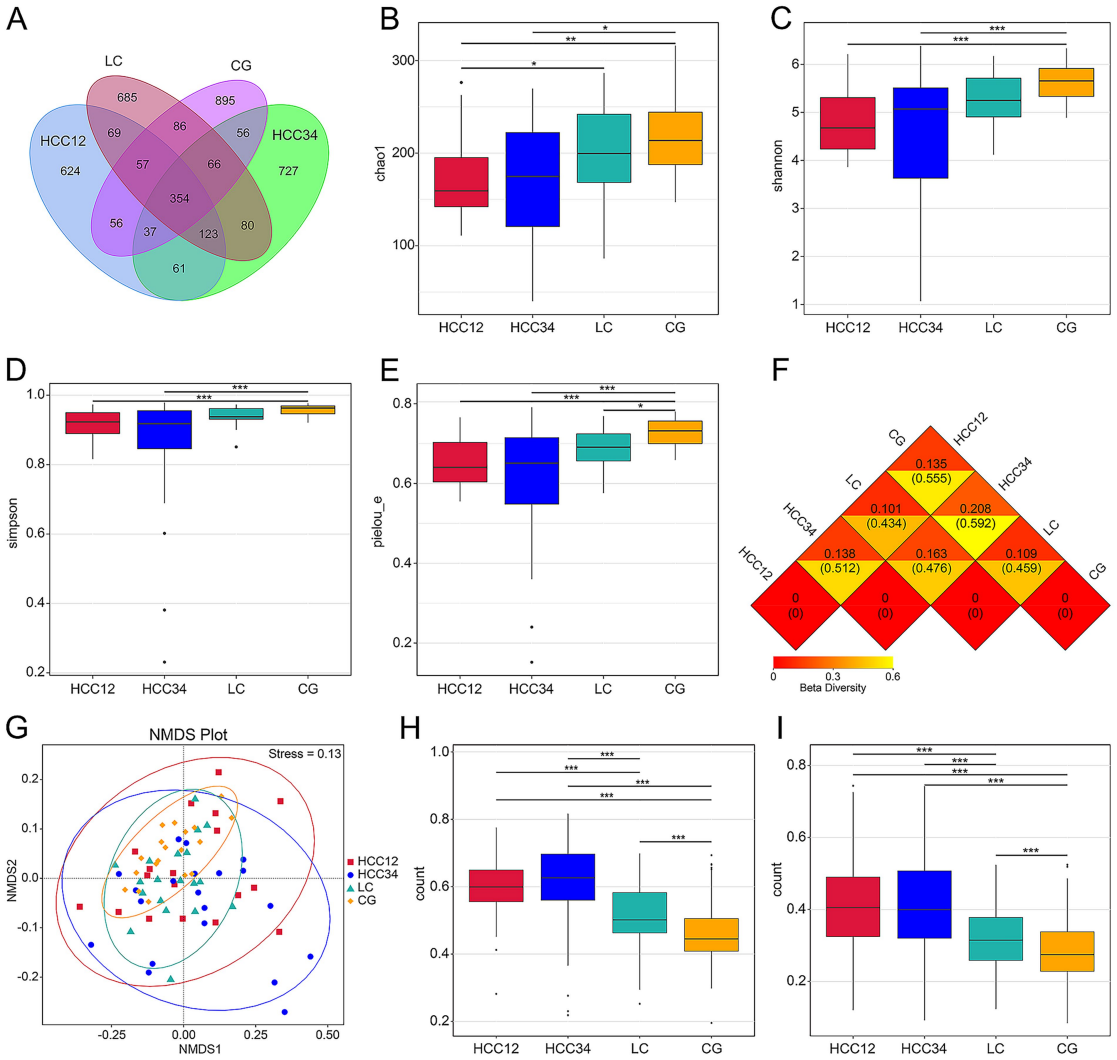
Groupwise comparisons of gut microbial α-diversity and β-diversity. **(A)** Venn diagram. **(B–E)** Comparisons of Chao1 richness, Shannon index, Simpson index, and Pielou’s evenness among the four groups. **(F)** Heatmap of the UniFrac distance matrix across groups. The upper and lower values within each square represent the weighted and unweighted UniFrac dissimilarity coefficients between samples, respectively; smaller coefficients indicate lower differences in microbial diversity between the corresponding samples. **(G)** NMDS analysis based on unweighted UniFrac distances among groups. Each point represents a sample, and the distances between points reflect differences in community structure (stress <0.2 indicates a reliable NMDS solution). **(H,I)** Intergroup differences were assessed using unweighted and weighted UniFrac Kruskal–Wallis tests. (^*^p < 0.05, ^**^p < 0.01, and ^***^p < 0.001).

Venn diagram analysis based on 16S rRNA amplicon sequencing revealed 624 and 727 unique ASVs in the HCC12 and HCC34 groups, respectively (Figure 1A). Gut microbial diversity appeared to play a role in distinguishing HCC patients. The α-diversity indices of both the HCC12 and HCC34 groups were significantly lower than those of healthy controls (Figures 1B–E). Specifically, analyses via the Chao1, Shannon, Simpson, and Pielou’s evenness indices indicated that both the diversity and abundance of the gut microbiota in HCC12 and HCC34 patients were reduced compared with the CG group. The Chao1 index further demonstrated that microbial richness in the HCC12 group was lower than that in the LC group. Pielou’s evenness index showed that species evenness in the LC group was decreased relative to the control group. Although the HCC34 group did not differ significantly from the LC group, all α-diversity indices were lower than those in the LC group.

The distance matrix heatmap showed that the HCC34 group exhibited the greatest dissimilarity with the CG group, whereas the HCC12 group showed the smallest dissimilarity with the LC group (Figure 1F). NMDS analysis indicated that samples from the CG group were tightly clustered, while the HCC12 and HCC34 groups were more dispersed, and the LC group overlapped with the other three groups (Figure 1G). Statistical analysis using unweighted and weighted UniFrac Kruskal–Wallis tests revealed that β-diversity differed significantly between the CG group and the other groups, as well as between the LC group and the other groups; however, no significant differences were observed between the HCC12 and HCC34 groups (Figures 1H,I).

### 3.3 Dominant gut microbial composition in advanced HCC patients

**Figure 2:**
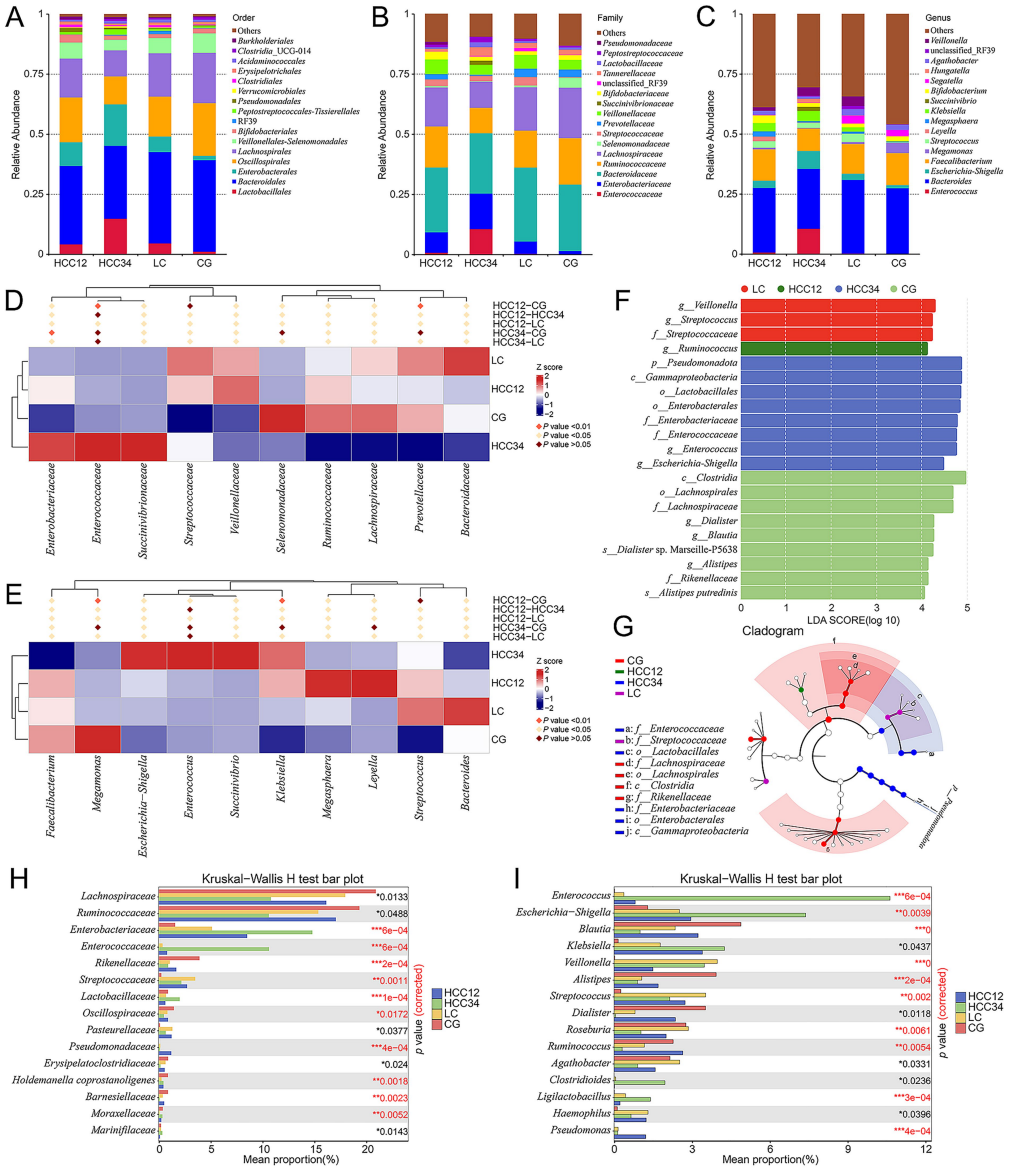
Species differences among groups. **(A–C)** Bar plots showing the top 15 most relatively abundant taxa at the order, family, and genus levels. **(D,E)** Differential families and genera (top 10) identified using zero-inflated negative binomial (ZINB) regression. The upper panels display clustering patterns and intergroup significance, and the heatmaps present *Z*-scores (standard scores). **(F)** Differential taxa among groups identified by LEfSe analysis (LDA score >4, *p* < 0.05). **(G)** Cladogram illustrating the phylogenetic relationships of the differential taxa. **(H,I)** Differential taxa at the family and genus levels (top 15) identified using the Kruskal–Wallis test. The *p*-values are shown on the right, with those highlighted in red indicating significance after FDR correction.

Analysis of the top 15 taxa at the order, family, and genus levels showed that the relative abundances of *Enterobacterales*, *Enterobacteriaceae*, and *Escherichia-Shigella* (the old NCBI hierarchical classification) progressively increased from the LC to HCC12 and HCC34 groups, reaching their highest levels in the HCC34 group (Figures 2A–C). Furthermore, the relative abundances of *Lactobacillales*, *Enterococcaceae*, and *Enterococcus* were also elevated in the HCC34 group (Figures 2A–C). This trend indicates a stepwise enrichment of specific gut microbiota as liver disease progresses from cirrhosis to HCC.

To identify taxa with significant differences between groups, we applied multiple statistical approaches. At the genus level, *Escherichia-Shigella*, *Enterococcus*, and *Succinivibrio*, and at the family level, *Enterobacteriaceae*, *Enterococcaceae*, and *Succinivibrionaceae* were dominant in the HCC34 group, with *Enterococcus* and *Enterococcaceae* showing particularly pronounced enrichment (Figures 2D,E). LEfSe analysis further identified four significantly enriched taxa across the groups (LDA score >4, *p* < 0.05). Notably, taxa within the same evolutionary lineage, *Enterobacterales*, *Enterobacteriaceae*, and *Escherichia-Shigella*, were enriched in HCC34, as were *Enterococcaceae* and *Enterococcus* (Figures 2F,G). The HCC12 group exhibited significant enrichment of *Ruminococcus*, whereas the LC group was dominated by *Streptococcaceae*, *Streptococcus*, and *Veillonella* (Figure 2F). Kruskal–Wallis rank-sum tests of family- and genus-level relative abundances yielded consistent results: *Enterobacteriaceae*, *Enterococcaceae*, *Lactobacillaceae*, *Escherichia-Shigella*, and *Enterococcus* were enriched in HCC34 (Figures 2H,I; Supplementary Figures S1A–E). Additionally, *Ruminococcus* was enriched in HCC12 and the control group but markedly reduced in HCC34, whereas *Veillonella* was elevated in both HCC34 and LC groups (Figure 2I; Supplementary Figures S1F,G).

We performed LEfSe analysis specifically within the HCC groups, which revealed that *Enterococcus*, *Bacilli*, *Lactobacillales*, and *Enterococcaceae* were significantly associated with distinguishing the HCC34 group (LDA score >4, *p* < 0.05) (Supplementary Figures S2A,B).

### 3.4 Associations between representative microbiota and clinical features

**Figure 3:**
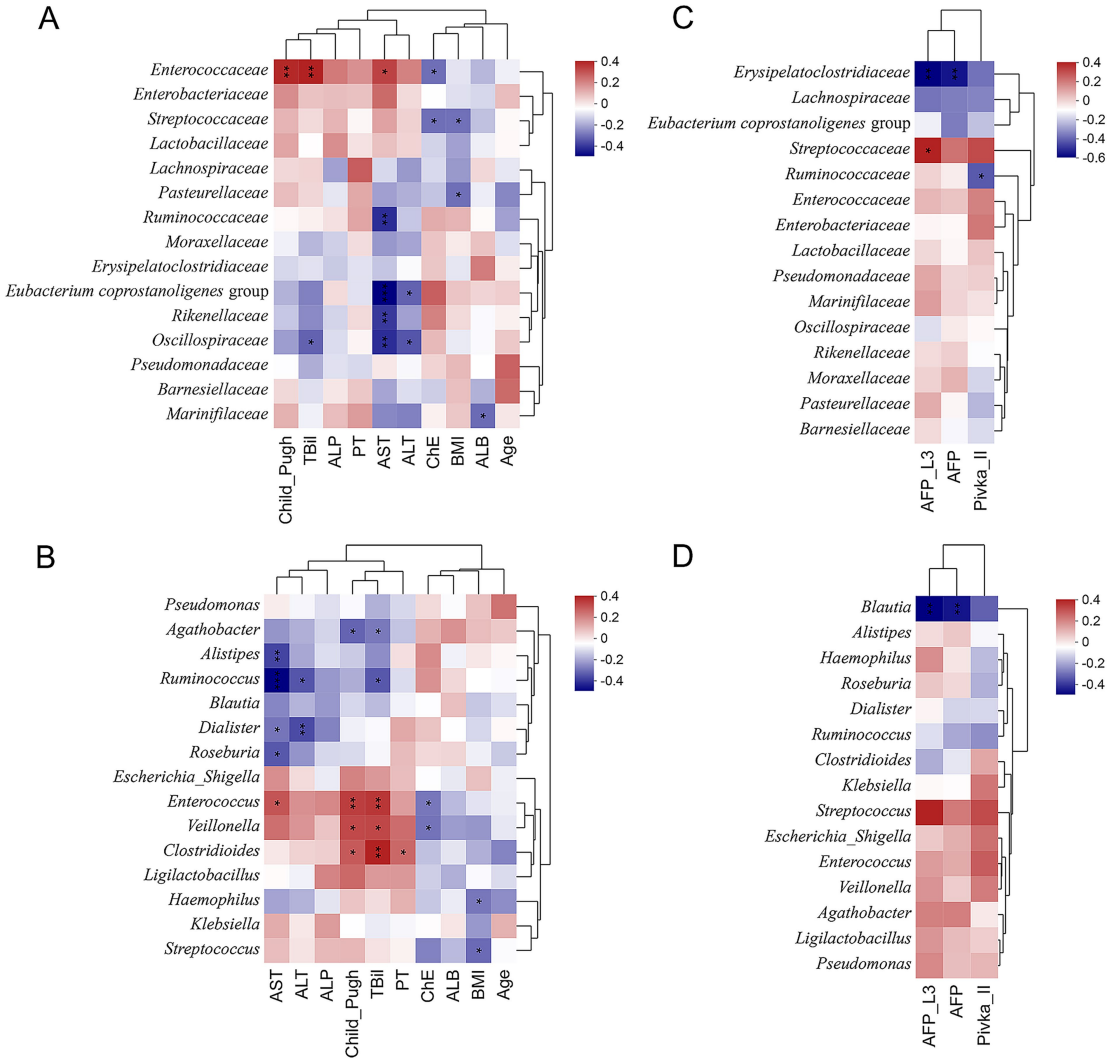
Spearman correlation heatmaps between clinical features and the top 15 gut microbial taxa identified by the Kruskal–Wallis test. **(A)** Correlations between liver function–related clinical features and gut microbiota at the family level. **(B)** Correlations between liver function–related clinical features and gut microbiota at the genus level. **(C)** Correlations between HCC-associated clinical characteristics and gut microbiota at the family level. **(D)** Correlations between HCC-associated clinical characteristics and gut microbiota at the genus level (^*^p < 0.05 and ^**^p < 0.01).

We examined the correlations between the top 15 family- and genus-level taxa identified by Kruskal–Wallis rank-sum tests and clinical features. At the family level (Figure 3A), *Enterococcaceae*, *Enterobacteriaceae*, *Streptococcaceae*, and *Lactobacillaceae* were positively correlated with ALT, AST, prothrombin time (PT), alkaline phosphatase (ALP), TBil, and Child–Pugh scores, with *Enterococcaceae* showing the strongest associations. In contrast, *Eubacterium coprostanoligenes* group, *Rikenellaceae*, and *Oscillospiraceae* were negatively correlated with ALT, AST, TBil, and Child–Pugh scores. These findings indicate that liver function impairment is accompanied by a relative decrease in *Eubacterium coprostanoligenes* group, *Rikenellaceae*, and *Oscillospiraceae*, and a relative increase in *Enterococcaceae*, *Enterobacteriaceae*, *Streptococcaceae*, and *Lactobacillaceae*. Conversely, ChE levels were significantly negatively correlated with *Enterococcaceae* and *Streptococcaceae*, and positively correlated with *Ruminococcaceae*, *Moraxellaceae*, *Erysipelatoclostridiaceae*, *Eubacterium coprostanoligenes* group, *Rikenellaceae*, *Oscillospiraceae*, and *Pseudomonadaceae*. Body mass index (BMI) exhibited a trend of negative association with liver cell injury, consistent with disease progression and malnutrition-related wasting.

At the genus level (Figure 3B), *Agathobacter*, *Alistipes*, *Ruminococcus*, *Blautia*, *Dialister*, and *Roseburia* were negatively correlated with liver injury-related clinical parameters, including TBil, Child–Pugh scores, ALP, ALT, and AST. In contrast, *Enterococcus*, *Veillonella*, and *Clostridioides* showed positive correlations with these clinical features. These findings suggest that *Enterococcus*, *Veillonella*, and *Clostridioides*, together with their corresponding families (*Enterococcaceae*, *Enterobacteriaceae*, *Streptococcaceae*, and *Lactobacillaceae*), are associated with the severity of liver injury.

In addition, *Erysipelatoclostridiaceae*, *Lachnospiraceae*, and *Blautia* were negatively correlated with the HCC biomarkers Pivka II, AFP, and AFP-L3 (Figure 3C), which may be related to treatment responses in HCC. Conversely, *Streptococcaceae* and *Streptococcus* showed positive correlations with these biomarkers (Figure 3D), suggesting the potential diagnostic value of *Streptococcus* in HCC. *Ruminococcaceae* exhibited a notably negative association with Pivka II (Figure 3C), a biomarker with high specificity for diagnosing HCC, indicating that *Ruminococcaceae* may also possess diagnostic potential for hepatocellular carcinoma.

### 3.5 Construction of a classification model for advanced HCC and identification of core microbial biomarkers

**Figure 4:**
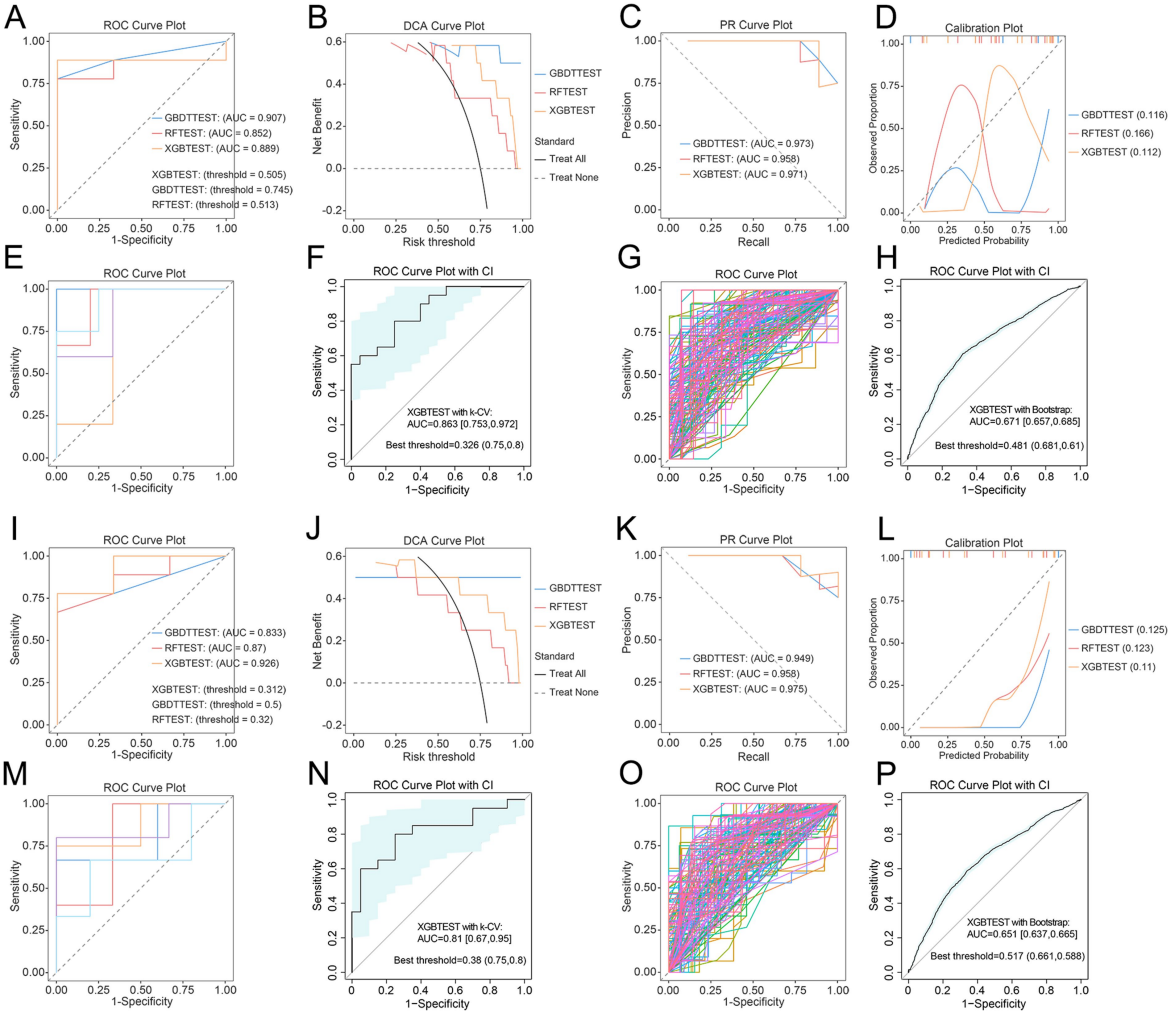
Construction of machine learning classification models for advanced HCC based on clinical features and representative microbiota. **(A–D)** ROC curves, decision curve analysis (DCA), precision-recall (PR) curves, and calibration plots for RF, GBDT, and XGB models constructed using clinical features. **(E,F)** *k*-fold CV of the XGB model based on clinical features. **(G,H)** Bootstrap validation of the XGB model based on clinical features. **(I–L)** ROC curves, DCA, PR curves, and calibration plots for RF, GBDT, and XGB models constructed using representative microbiota identified by the Kruskal–Wallis test. **(M,N)** *k*-fold CV of the XGB model based on representative microbiota. **(O,P)** Bootstrap validation of the XGB model based on representative microbiota.

**Figure 5:**
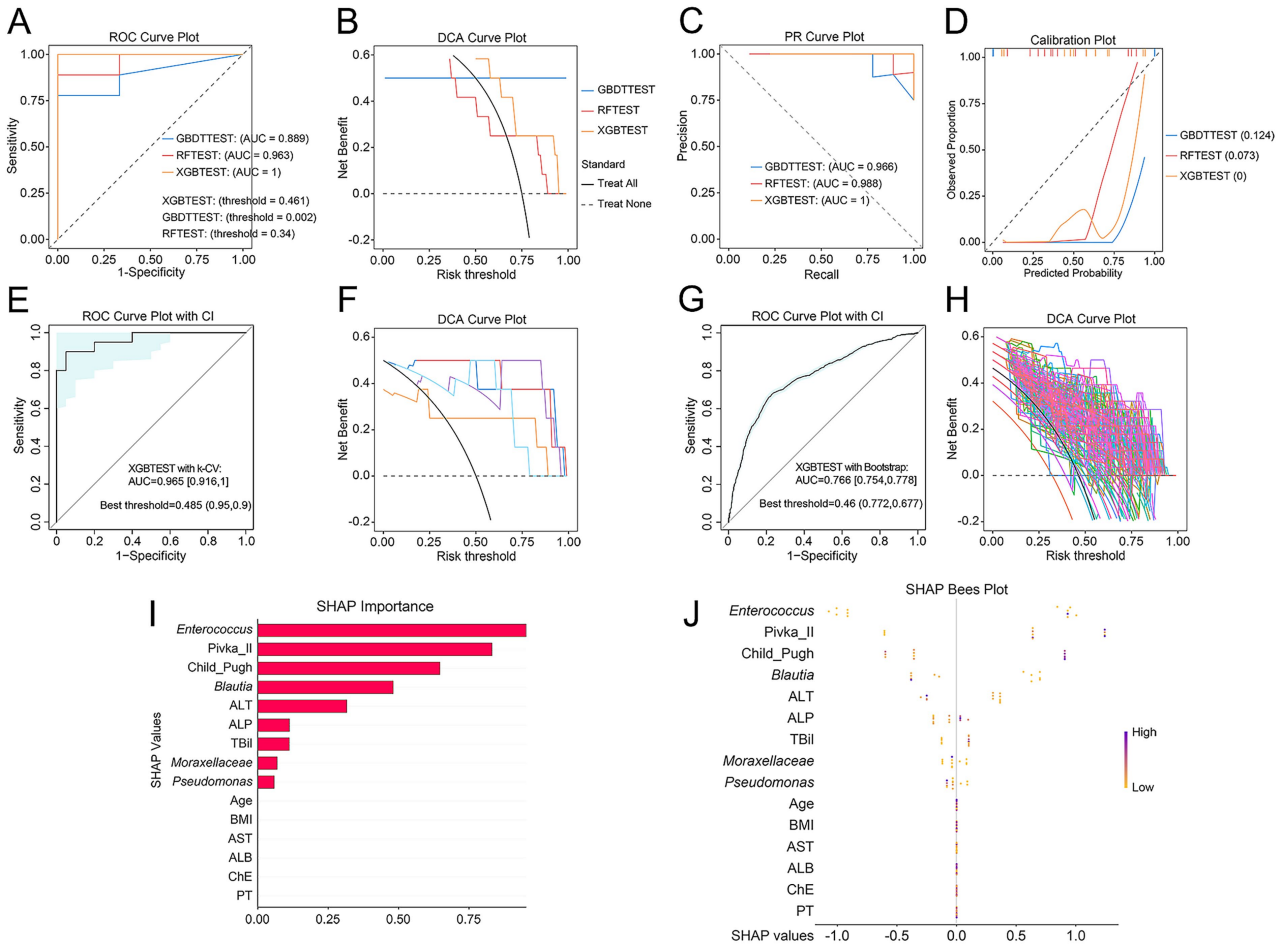
Construction of machine learning classification models for advanced HCC based on clinical features combined with representative microbiota. **(A–D)** ROC curves, DCA, PR curves, and calibration plots for RF, GBDT, and XGB models. **(E,F)** *k*-fold CV of the XGB model. **(G,H)** Bootstrap validation of the XGB model. **(I)** SHAP bar plot displaying the importance ranking of feature variables in discriminating advanced HCC. **(J)** SHAP bees plot illustrating the distribution of SHAP values for each feature; each dot represents the SHAP value of a given feature in an individual sample, with color indicating the feature value.

Among the 38 patients with HCC, we first constructed machine learning classification models for advanced HCC using clinical variables, including RF, GBDT, and XGB. The XGB model demonstrated the best performance (AUC = 0.889) (Figures 4A–H). We then developed models using the top 15 family- and genus-level microbial taxa identified by Kruskal–Wallis rank-sum tests. The XGB model based solely on microbial features showed strong discriminatory ability for identifying HCC34, with the highest performance (AUC = 0.926) (Figures 4I–P). Finally, integrating clinical features with key microbial taxa further improved the diagnostic performance for HCC34, achieving optimal discrimination (AUC = 1) (Figures 5A–D). To minimize overfitting, we applied both bootstrap resampling and *k*-fold CV for internal validation of each model. The results indicated that differential microbial taxa possess diagnostic value for HCC staging, and that combining clinical variables with microbial features yields the best performance (XGB with bootstrap: AUC = 0.943; XGB with *k*-fold CV: AUC = 0.766) (Figures 5E–H).

Feature importance analysis revealed that *Enterococcus*, Pivka II, Child–Pugh scores, *Blautia*, ALT, ALP, TBil, *Moraxellaceae*, and *Pseudomonas* were key contributors to distinguishing HCC34 from HCC12 (Figures 5I,J). Except for ALT, the remaining eight features were positively associated with HCC34. Notably, PIVKA-II and Child–Pugh scores are integral components of the CNLC staging system (Zhou et al., 2025). *Enterococcus*, *Moraxellaceae*, and *Pseudomonas* were specifically enriched in the HCC34 group, consistent with the earlier differential abundance analyses. Among them, *Enterococcus* showed the strongest discriminatory value for differentiating HCC34 from HCC12.

**Figure 6:**
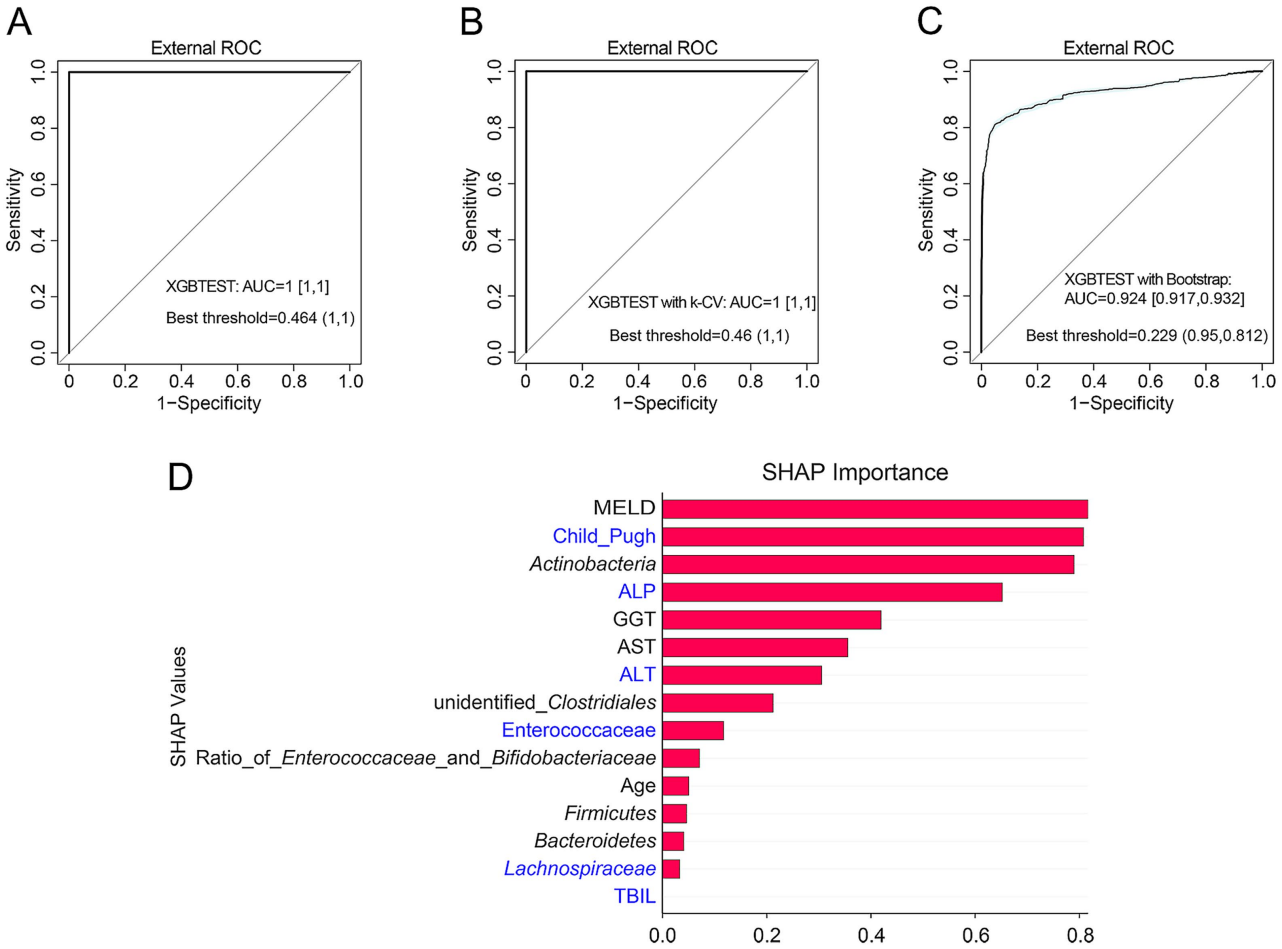
External validation of XGB models for advanced HCC. **(A)** ROC curves of the XGB model evaluated using the external dataset. **(B)** *k*-fold CV of the XGB model based on the external dataset. **(C)** Bootstrap validation of the XGB model based on the external dataset. **(D)** SHAP bar plot based on the external dataset, displaying the importance ranking of feature variables in discriminating advanced HCC. Features highlighted in blue represent key variables consistently identified as important in our study cohort.

Using the clinical characteristics and key microbial taxa reported by Zhang et al. (2021) for 74 patients with HCC, we reclassified their early and intermediate groups as HCC12 and their terminal group as HCC34 (Supplementary Table S1). External validation using our optimized XGB model demonstrated that the top nine features selected by SHAP (Figure 5I) showed excellent discriminatory performance for advanced HCC (AUC = 1) (Figure 6A). Independent validation with bootstrap resampling and *k*-fold CV also yielded robust results (bootstrap: AUC = 0.924; k-fold CV: AUC = 1) (Figures 6B,C). Moreover, the feature importance ranking derived from the XGB model, which highlighting Child–Pugh scores, ALP, ALT, *Enterococcaceae*, and *Lachnospiraceae*, was consistent with the key features identified in our cohort (Figure 6D). Evaluation metrics for all models are provided in Supplementary Table S2.

### 3.6 Functional prediction of the gut microbiome in advanced HCC

Based on *t* test analyses of microbiome functional predictions, the HCC34 group exhibited enrichment of peptidases, glutathione metabolism, and K03564 (thioredoxin-dependent peroxiredoxin) compared with the CG group (Supplementary Table S3). The enhanced peptidase activity may reflect the hypermetabolic and catabolic state characteristic of advanced HCC, in which increased proteolysis accelerates the breakdown of luminal proteins. The upregulation of glutathione metabolism and thioredoxin-dependent peroxiredoxin suggests pronounced oxidative stress within the gut microenvironment. Glutathione represents a major endogenous antioxidant, while K03564 is crucial for detoxifying peroxides. Their concurrent elevation indicates increased levels of reactive oxygen species (ROS), triggering peroxiredoxin-mediated peroxide removal and compensatory activation of glutathione metabolism. This oxidative stress–driven feedback loop may further exacerbate disease progression in advanced HCC.

## 4 Discussion

This study systematically characterized the gut microbiota of healthy adults, patients with liver cirrhosis, and HCC patients at different stages using 16S rRNA amplicon sequencing. From a microbiological perspective, we identified microbial taxa enriched in advanced HCC and, by integrating clinical features, developed a highly effective machine learning model. These findings provide novel insights and potential biomarkers for the precise identification of advanced HCC.

This study, from multiple perspectives, for the first time suggests the potential diagnostic relevance of *Enterococcus* in advanced HCC. *Enterococcus* was consistently identified as significant across various statistical analyses, including Kruskal–Wallis tests, LEfSe, and ZINB, in agreement with previous studies (Liu et al., 2019; Ponziani et al., 2019; Iida et al., 2021). Machine learning results further indicated that *Enterococcus* is one of the most critical features for distinguishing advanced HCC. Notably, a classification model based solely on gut microbial features achieved high discriminatory performance for advanced HCC, which was further improved when combined with clinical indicators. *Enterococcus*, together with PIVKA II and Child–Pugh scores, emerged as key discriminative features for advanced HCC. Moreover, *Enterococcus* was positively correlated with liver injury markers such as ALT, TBil, and Child–Pugh scores, as well as with HCC-specific biomarkers including PIVKA II and AFP-L3. These findings underscore the potential diagnostic value of *Enterococcus* in advanced HCC. Integration of *Enterococcus* with the existing GALAD model, which includes gender, age, AFP, PIVKA-II, and AFP-L3, may enhance diagnostic accuracy, particularly offering a simpler, noninvasive approach for detecting late-stage liver cancer (Huang et al., 2022).

*Enterococcus* is a common commensal bacterium in the human gut, but under conditions of gut dysbiosis, it can induce inflammatory responses by activating the toll-like receptor 4/nuclear factor-κB (TLR4/NF-κB) pathway, thereby promoting the progression of chronic liver disease to HCC (Seki et al., 2007; Iida et al., 2021). From a biodiversity perspective, gut microbial diversity is significantly reduced in HCC patients, particularly in advanced stages, reflecting a gradual depletion of microbiota and progressive dysbiosis along the hepatitis–cirrhosis–HCC continuum (Trebicka et al., 2021). This observation aligns with the “gut–liver axis” concept, whereby impaired liver function, increased portal vein pressure, and intestinal barrier disruption collectively drive escalating microbial imbalance, thereby facilitating HCC initiation and progression (Tripathi et al., 2018; Hsu and Schnabl, 2023; Li et al., 2025). Dysbiosis is also accompanied by enrichment of pathogenic bacteria and altered metabolites, such as increased LPS, which can modulate immune responses and trigger inflammation (Dapito et al., 2012). Hepatic cells, including Kupffer cells, hepatic stellate cells (HSCs), and hepatocytes, express the pattern recognition receptor TLR4, which specifically recognizes gut-derived LPS. Binding of LPS to TLR4 activates downstream signaling pathways; in HSCs, TLR4 activation can promote disruption of hepatocyte apoptosis mediated by NF-κB, ultimately facilitating HCC development (Schwabe and Greten, 2020; Li et al., 2022). This mechanistic insight is consistent with our functional predictions, which revealed pronounced activation of oxidative stress responses in the gut microbiome of advanced HCC. Specifically, peptidase activity, glutathione metabolism, and thioredoxin-dependent peroxiredoxin functions were significantly elevated in the HCC34 group, suggesting that the gut microbiota in advanced HCC may be associated with inflammatory regulation and antioxidant stress adaptation. These functional alterations further suggest that specific microbial taxa in advanced HCC may be associated with tumor progression and provide a theoretical basis for exploring microbiota-targeted metabolic interventions.

In contrast, bacteria producing SCFAs, such as *Ruminococcus*, *Blautia*, and *Dialister*, were enriched in the gut microbiota of early-stage HCC and control groups, while *Alistipes* was more abundant in healthy individuals. These microbes help maintain intestinal barrier integrity, regulate immune metabolism, and suppress inflammatory responses. Their reduction may indicate impaired gut defense mechanisms and represents a key feature of microbial dysbiosis in advanced tumor stages (Liu et al., 2019; Zhang et al., 2019; Bi et al., 2021; Medawar et al., 2021). The enrichment of *Ruminococcus*, *Blautia*, and *Dialister* in early HCC may be associated with a compensatory defensive response. As the disease progresses, the abundance of beneficial bacteria sharply declines in advanced HCC, whereas pathogenic bacteria become highly enriched, accompanied by the activation of oxidative stress responses. These changes may be associated with HCC progression. Therefore, accurate identification of advanced HCC and early correction of gut microbial dysbiosis could potentially be beneficial for disease management.

This study collected a real-world clinical cohort and combined microbiome analysis with machine learning models to focus on the gut microbiota of advanced HCC, providing preliminary evidence for its accurate diagnosis. However, several limitations should be acknowledged. First, although multiple internal and external validation strategies were applied, the relatively limited sample size may increase the potential risk of model overfitting. Therefore, the extremely high AUC values observed in this study should be interpreted with caution and regarded as proof-of-concept findings rather than clinically validated diagnostic assays. Prospective, large-scale, multicenter studies will be essential to further assess the robustness and translational potential of these microbiome-based models before their application in routine clinical practice. Second, the machine learning model for advanced HCC was externally validated using data from a single published study with a relatively limited sample size. Due to differences in taxonomic resolution, several microbial taxa were unavailable and were therefore substituted with higher-level taxonomic categories, and missing values for three features were imputed using the mean values from the training cohort. Although this approach allowed for exploratory external validation, it may have introduced classification bias and potentially inflated model performance. Consequently, the external validation results should be interpreted cautiously and warrant further confirmation in independent datasets with consistent taxonomic resolution. Third, 16S rRNA sequencing cannot resolve microbial taxa at the strain level or fully characterize metabolic functions, highlighting the need for complementary metagenomic and metabolomic analyses. Developing animal models and conducting *in vitro* experiments to elucidate the causal mechanisms linking core taxa (such as *Enterococcus*) to HCC will be essential for further investigation. In addition, precise strain-level quantification of *Enterococcus* using digital PCR may further enhance the diagnostic accuracy for advanced HCC.

Overall, this study provides insights into the potential associative role of the gut microbiota in HCC progression and suggests that combining *Enterococcus* with clinical features may offer noninvasive diagnostic value for identifying advanced HCC. In addition, it proposes that correcting gut dysbiosis might serve as a potential adjunctive therapeutic strategy. These findings provide exploratory targets and preliminary insights for the noninvasive diagnosis and precision management of advanced HCC.

## Data Availability

The original contributions presented in the study are publicly available. This data can be found at the NCBI Sequence Read Archive: https://www.ncbi.nlm.nih.gov/, accession number PRJNA1397951.
