# Supplementary material for: Gut microbial signatures of advanced hepatocellular carcinoma and their potential diagnostic value
Source: Front Microbiol. 2026 Feb 2;17:1760859. doi: 10.3389/fmicb.2026.1760859 (PMC12908660; doi:10.3389/fmicb.2026.1760859)
Supplement: Supplementary file 2 [file Data_Sheet_1.docx]

**Supplementary Figures**


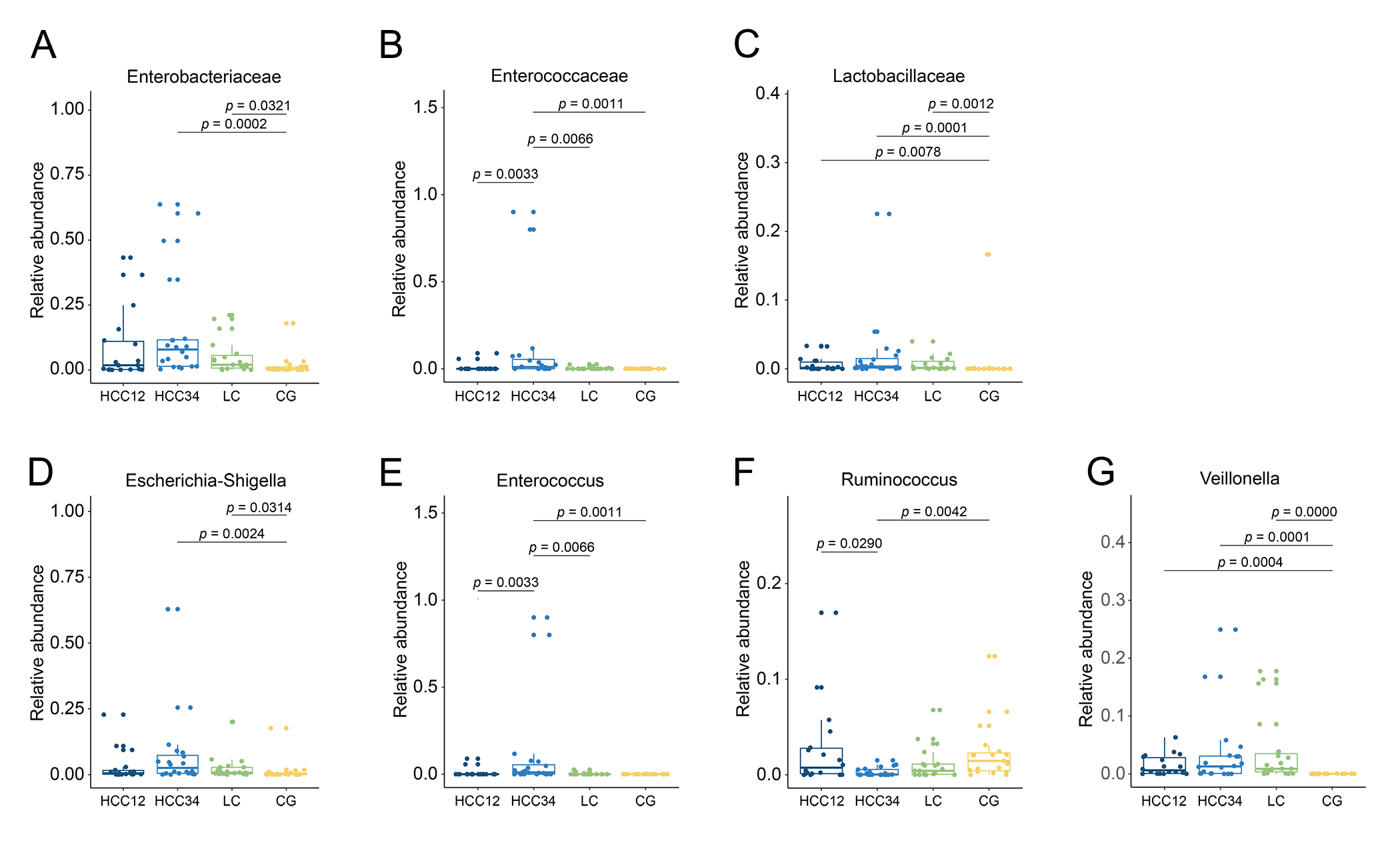


**Figure S1*.*** Dunn’s post hoc test evaluating differences in the relative abundances of key microbiota among the four groups (HCC12, HCC34, LC, and CG). The annotated *p* values represent results after FDR correction.


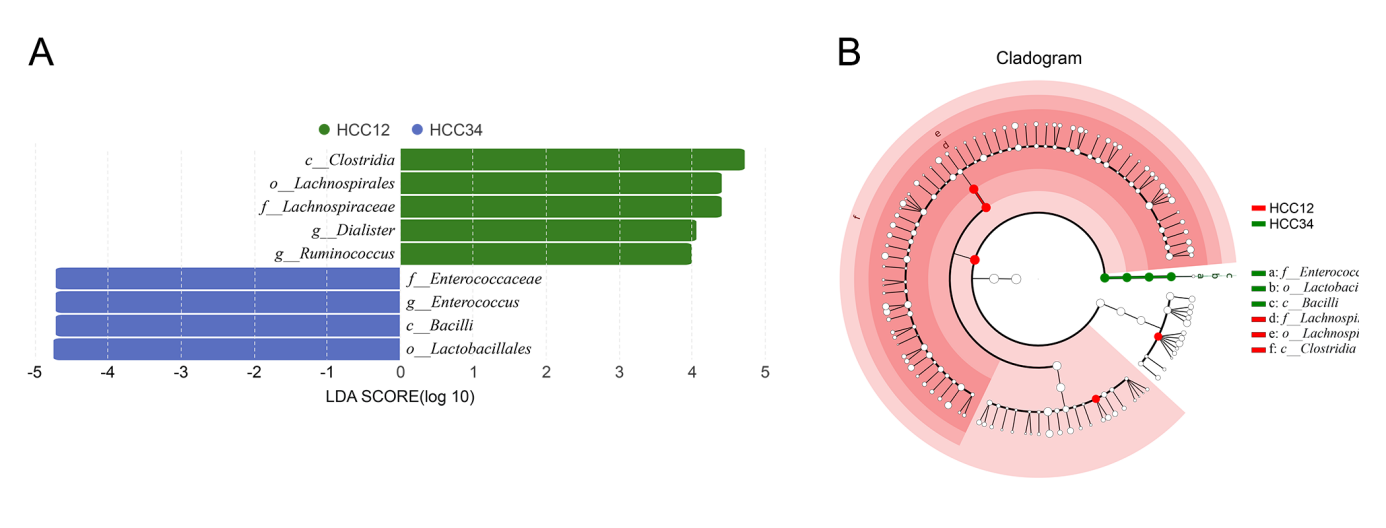


**Figure S2.** Differential taxa among HCC12 and HCC34 groups identified by LEfSe analysis (LDA score > 4, *p* < 0.05). (G) Cladogram illustrating the phylogenetic relationships of the differential taxa.
